# Supplementary material for: Deletion of wfs1 Impairs Oligodendrocyte Precursor Cells Dorsal Distribution and Myelination Through the wfs1-hmgcs1 Axis in Zebrafish
Source: Biology (Basel). 2026 Aug 16;15(16):1402. doi: 10.3390/biology15161402 (PMC13510151; doi:10.3390/biology15161402)
Supplement: Supplementary file 1 [file biology-15-01402-s001.zip › File S1-Transcriptome sequencing data and differentially expressed genes (DEGs) in wfs1-- zebrafish at 5 dpf/2.KEGG/wfs1KOvsWT/DOWN/src/dre00500.html]

dre00500


- dre:406339

- Down regulated genes

406339\_1(-1.38680349052238)

- dre:561803

- Down regulated genes

561803(-1.33365257250416)

- dre:406339

- Down regulated genes

406339\_1(-1.38680349052238)

- dre:100333665

- Down regulated genes

100333665\_1(-1.77123973220434)

Close
